# Editorial: Global excellence in bacteriology: Central and South America

**DOI:** 10.3389/fcimb.2025.1563952

**Published:** 2025-02-13

**Authors:** Fernando Navarro-Garcia, Marcelo Brocchi, Angel A. Cataldi

**Affiliations:** ^1^ Department of Cell Biology, Centro de Investigación y Estudios Avanzados, México City, Mexico; ^2^ Departamento de Genética e Evolução, Microbiologia e Imunologia, Instituto de Biologia, Universidade Estadual de Campinas, Campinas, Brazil; ^3^ Instituto de Agrobiotecnología y Biología Molecular (IABIMO) Instituto Nacional de Tecnología Agropecuaria- Concejo Nacional de Investigaciones Científicas y Técnicas (INTA-CONICET), Hurlingham, Argentina

**Keywords:** antimicrobial resistance, complement system lysis, mucus hypersecretion, bacterial transmission, bacteriophage therapy

Bacteria are a support system for all life forms on the planet and are one of the ancestors of all living beings. Paradoxically, some bacteria are a threat to human, plant and animal health. Because of their importance, this is an exciting time for bacteriology. Bacteriological research is changing rapidly in response to the emergence of diseases of global importance, the threat of bioterrorism, and the increasing failure of antibiotics and therapies. Areas that are especially conducive to significant progress include genomics and basic areas of biological complexity (infectious diseases and the engineering of bacteria designed for the benefit of society), along with the evolution and ecology of microorganisms. In this context, this Journal has organized a series of Research Topics to highlight the latest advances in bacteriology around the world. Thus, the Research Topic *Global Excellence in Bacteriology in Central and South America*, includes a series of five publications on different topics, such as antimicrobial resistance, bacteriophage as phage therapy, virulence factors to overcome the host immune system (complement system or intestinal mucus layer), and bacterial transmissibility mechanism.

Antimicrobial resistance (AMR) is a global public health problem that threatens the effectiveness of medical treatments and patient safety. Overuse and inappropriate use of antimicrobials in human/veterinary medicine contribute to the selection and dissemination of resistant microorganisms. Understanding the mechanisms and pathways of AMR dissemination is essential to identify the microbial genes involved and to develop effective strategies to prevent and control spread. Thereby, Carrera Páez et al. addressed the scarcely studied role of sporadic bacterial clones in AMR dissemination. The authors used a sporadic clone strain, isolated in South America, that carries the mcr-1 gene. mcr-1 encodes resistance to colistin, one of the last barriers to overcoming AMR. Carrera-Páez et al. showed that the sporadic clone can acquire and transfer different types of β-lactamase genes, Importantly, these AMR genes can be carried by extracellular vesicles. Therefore, although sporadic or orphan clones are epidemiologically unsuccessful, they can be efficient receptors and donors of foreign DNA and thus be a reservoir of antimicrobial resistance genes.

Bacteriophages are viruses that infect and kill bacteria. There are two main groups, lysogenic and lytic phages. Bacteriophages that infect human pathogens are considered potential biocontrol agents, and studying their genetic content is essential for their safe use in the food industry and antimicrobial therapy. Soto Lopez et al. characterized the genome, physiology and morphology of the bacteriophage *Tequatrovirus ufvareg1*, to which *Escherichia coli* O157:H7 is susceptible. Through comparative genomics and phylogenetic analysis, the authors showed the degree of conservation between *T. ufvareg1* and the genus *Tequatrovirus*. Furthermore, the authors demonstrated the ability of this virus to lyse *E. coli* O157:H7 and determined the structural morphology of the phage. Based on the annotated genome Soto Lopez et al. did not find any virulence-related genes or integrases. The lack of virulence genes (i.e. toxin genes) the fact that *T. ufvareg1 * is not an integrative lysogenic bacteriophage are desirable characteristics for a bacteriophage formulation that can be used in phage therapy and biocontrol. Therefore, *ufvareg1* would be a promising, effective and safe candidate for the control of food-borne pathogenic *E. coli*.

The complement system (CS) is an important defense mechanism of the immune system that can be activated by invading pathogens. Proteases produced by pathogenic bacteria, such as SPATEs, can cleave CS components and promote bacterial resistance to human serum. Considering the presence of the *pet* gene (a SPATE) in *E. coli* strains that cause extraintestinal infections, Correa et al. investigated whether Pet can also contribute to serum resistance *in vitro*. The authors found that Pet, but not Pet S260I (a catalytic site mutant), can potentially interfere with the alternative pathway of CS activation and the formation of important by-products by cleaving key components of the cascade. Additionally, Pet can also inactivate the terminal pathway by targeting C9, thereby preventing C9 polymerization and lytic pore formation; inhibition of C9 polymerization protected the *E. coli* DH5α strain from lysis by human serum. Since the ability to evade lysis by the CS facilitates bacterial survival in the bloodstream, Pet may also play an important role in the pathogenesis of sepsis caused by *E. coli*.

Some Gram-negative and Gram-positive bacterial factors induce mucus production in the host. Previous data showed that two atypical EPEC (aEPEC) strains have the unique ability to increase mucus production and secretion in an *in vivo* ligated rabbit ileal loop (LRIL) model. However, the prototype (E2348/69) tEPEC (typical strains carry the pEAF plasmid, which aEPEC lacks) did not induce this phenomenon. Trovão et al. further studied the mechanisms involved in the increased mucus production induced by aEPEC strains when tested in the LRIL model. Genomic analyses showed two additional aEPEC strains belonging to the same mucus-inducing cluster, which were identified as mucus-inducing strains. Further analysis showed that all 13 strains were able to use mucin as a carbon source, 10 strains were able to pass through a mucin layer, and 4 were better at adhering to mucin-agar. A large diversity of virulence factors was detected in the strains, confirming the genomic heterogeneity that characterizes the aEPEC group and explaining the absence of a clear association between genes, groups of genes, and the mucin-inducing phenotype. These data provide important new information on the mechanisms involved in the pathogenesis of aEPEC.

A member of the *Mycobacterium tuberculosis* complex (MTC) is *Mycobacterium pinnipedii* (Mp); the causative agent of tuberculosis in pinnipeds (i.e. seals). Mp can also infect non-marine mammals, including humans. Since the transmissibility of a pathogen is related to its virulence, Marfil et al. investigated the transmissibility of one Mp strain and two *M. bovis* strains (Mb, with different virulence profiles) in a mouse model. Uninoculated (sentinel) mice were housed together with intratracheally inoculated mice. A 100% transmissibility was observed at 30 dpi in sentinel mice housed with Mp- and Mb-inoculated mice (high virulence), as demonstrated by the recovery of viable Mp and Mb from the lungs of the sentinel mice. Mice inoculated with Mp and Mb (low virulence) survived until they were sacrificed, whereas those inoculated with Mb (high virulence) died at 17 dpi. Marfil et al. highlight the importance of the transmissibility of Mp, as a zoonotic agent, and the usefulness of the experimental model to study virulence characteristics such as bacterial transmission.

Thus, this Research Topic achieves the objective of highlighting the latest advances in bacteriology in the Central and South American regions, showcasing academic excellence and high-quality work by internationally recognized researchers.

